# The Impact of Maternal Obesity and Excessive Gestational Weight Gain on Maternal and Infant Outcomes in Maine: Analysis of Pregnancy Risk Assessment Monitoring System Results from 2000 to 2010

**DOI:** 10.1155/2016/5871313

**Published:** 2016-09-22

**Authors:** Nancy Baugh, David E. Harris, AbouEl-Makarim Aboueissa, Cheryl Sarton, Erika Lichter

**Affiliations:** ^1^Department of Nursing, Franklin Pierce University, Portsmouth, NH 03801, USA; ^2^School of Nursing, University of Southern Maine, Portland, ME 04104, USA; ^3^Department of Mathematics and Statistics, University of Southern Maine, Portland, ME 04104, USA; ^4^Department of Applied Medical Sciences, University of Southern Maine, Portland, ME 04104, USA

## Abstract

The objective of this study is to understand the relationships between prepregnancy obesity and excessive gestational weight gain (GWG) and adverse maternal and fetal outcomes. Pregnancy risk assessment monitoring system (PRAMS) data from Maine for 2000–2010 were used to determine associations between demographic, socioeconomic, and health behavioral variables and maternal and infant outcomes. Multivariate logistic regression analysis was performed on the independent variables of age, race, smoking, previous live births, marital status, education, BMI, income, rurality, alcohol use, and GWG. Dependent variables included maternal hypertension, premature birth, birth weight, infant admission to the intensive care unit (ICU), and length of hospital stay of the infant. Excessive prepregnancy BMI and excessive GWG independently predicted maternal hypertension. A high prepregnancy BMI increased the risk of the infant being born prematurely, having a longer hospital stay, and having an excessive birth weight. Excessive GWG predicted a longer infant hospital stay and excessive birth weight. A low pregnancy BMI and a lower than recommended GWG were also associated with poor outcomes: prematurity, low birth weight, and an increased risk of the infant admitted to ICU. These findings support the importance of preconception care that promotes achievement of a healthy weight to enhance optimal reproductive outcomes.

## 1. Introduction

While the prevalence of obesity has recently stabilized in the United States, approximately 60 percent of women of reproductive age are either overweight or obese [1] and as a result are at increased risk for complications during pregnancy [2]. Morbidly obese women are at particularly high risk for pregnancy complications including gestational diabetes, hypertension, preeclampsia, asthma, and venous thromboembolism [3–7]. Obesity is an independent predictor of cesarean delivery, with the rate of cesarean delivery increasing proportionately with increasing Body Mass Index (BMI) [7]. Most concerning, obese women have a significantly higher risk of mortality during pregnancy and labor than women with a normal BMI [8].

The association between maternal obesity and adverse fetal outcomes such as preterm labor, congenital abnormalities, macrosomia, and shoulder dystocia is also well established [8]. Maternal obesity is associated with an increased risk of fetal death, stillbirth, neonatal death, perinatal death, and infant death [9–14]. The risk of fetal death after 20 weeks of gestation in obese women has been shown to increase in a dose-dependent fashion as weight increases [13].

While entering pregnancy overweight or obese increases the risk for pregnancy complications, excessive gestational weight gain (GWG) also increases the risk of adverse outcomes for the mother and infant. Current Institute of Medicine (IOM) guidelines recommend a total weight gain of 15–25 lbs during pregnancy for overweight women with a BMI between 25 and 29.9 [14]; however, a high proportion of women gain more weight than recommended [15]. Women who gain 28 lb by 28 weeks are at a much higher risk for induction of labor, cesarean delivery, wound infection, and infant shoulder dystocia [6]. Obese women who gain more than recommended weight are also at higher risk for preeclampsia, gestational diabetes, cesarean section, macrosomia, neonatal morbidity, and postpartum weight retention [15, 16].

Given the correlations between prepregnancy obesity and excessive maternal weight gain during pregnancy on the one hand and health challenges for both the pregnant woman and her infant on the other, it is important to determine how prepregnancy obesity and excessive gestational weight gain interact with other factors to place pregnancies at the greatest risks. To help address this question, we performed multivariable analysis on data for the State of Maine obtained from the PRAMS (Pregnancy Risk Assessment Monitoring System) project, a national database collected and maintained by the Centers for Disease Control and Prevention [17]. The PRAMS dataset is an ideal source for this analysis because it collects a wide range of information from women who have recently given birth to a live infant. Here we report correlational links between demographic, socioeconomic, and health behavioral independent variables including maternal prepregnancy obesity and excess pregnancy weight gain and dependent variables related to maternal complications during pregnancy and poor infant outcomes. These results will be of interest to practitioners who care for women before and during pregnancy, public health workers who seek to address health on a community level, and researchers who study the impact of obesity and weight gain on health. This question is of particular importance in Maine, a state where prepregnancy BMI increased steadily between 2000 and 2010 [18].

## 2. Methods

### 2.1. The PRAMS Dataset

This study analyzes data from the Maine Pregnancy Risk Assessment Monitoring System (PRAMS) questionnaires for the years 2000–2010, which we obtained from the Maine Department of Health and Human Services [17]. PRAMS is a national population-based survey tool developed by the Center for Disease Control and Prevention (CDC) [19] to monitor selected self-reported behaviors, healthcare use, and maternal morbidities that occur before, during, and after pregnancy. The PRAMS questionnaire provides data on self-reported maternal demographics, socioeconomic variables, prepregnancy health behaviors, health behaviors during pregnancy, and pregnancy outcomes (for both mother and newborn). These data are matched with additional information obtained from the birth certificates.

To compile this dataset, the Maine Department of Health and Human Services uses birth certificates to identify women who gave birth to a live infant within the previous 2–4 months. A stratified sample of 125 women per month is selected and mailed PRAMS questionnaires. Up to 2 follow-up questionnaires are mailed to nonresponders with attempted telephone follow-up during the subsequent 9 months of the postpartum period. Members of high-risk groups (e.g., women with low birth weight infants and Medicare recipients) were oversampled.

### 2.2. Variables Analyzed

To determine the maternal factors related to poor maternal and infant outcomes, we analyzed a broad range of variables from the PRAMS dataset. Maternal prepregnancy height and weight were used to calculate BMI, which was analyzed as a continuous variable. Maternal age as well as the gestational age when each woman was sure she was pregnant and at her first prenatal visit (in weeks) was also analyzed as continuous variables. The following variables were analyzed as dichotomous variables: previous live birth (yes or no), marital status (married or not married), educational attainment (≤12th grade or >12th grade), household income (<$20,000/year or >$20,000/year), rurality of residence (urban and suburban or small rural town and isolated rural location using RUCA codes), race (white or other), alcohol consumption before and in the last 3 months of pregnancy (yes or no), premature birth by dates (<37 weeks or 37–42 weeks), infant admission to an intensive care unit (ICU) (yes or no), and maternal hypertension during pregnancy (yes or no). The following variables were analyzed as categorical variables with >2 categories: tobacco consumption (never smoked, smoked before pregnancy only, or smoked before and during pregnancy), gestational weight gain (<recommended, within recommended range, or >recommended as defined by the Institute of Medicine based on prepregnancy obesity status [14] using within recommended range as the reference category), infant birth weight (<2500 gm, 2500–3999 gm, and ≥4000 gm) using 2500–3999 gm as the reference category, and length of infant hospital stay (1-2 days, 3–5 days, or ≥ 6 days). The birth weight ranges were chosen to match the National Institutes of Health definitions of low, normal, and excessive birth weight [20].

### 2.3. Data Analysis

We performed multivariable logistic regression analysis using the survey procedures in the Statistical Analysis Software v9.3 [21] to adjust for the complex sampling strategy (e.g., oversampling of at risk populations) of the PRAMS dataset. The independent variables we considered included smoking (before and during pregnancy), age, pervious live births, marital status, education, prepregnancy BMI, annual household income, urban versus rural residence, race, alcohol consumption (before and during pregnancy), gestational age when woman was sure she was pregnant and at first prenatal visit, and pregnancy weight gain. The dependent variables related to infant outcomes were infant admission to an intensive care unit, length of hospital stay, low and excessive birth weight, and premature birth. Maternal hypertension was an additional dependent variable. Adjusted odds ratios, 95% confidence intervals, and *P* values were calculated and significance was accepted at *P* < 0.05 level. The confidence intervals we report are 95% Wald Confidence Intervals for adjusted odds ratios. Thus the odds ratios for the independent variables in these multivariable analyses are adjusted for all the other independent variables in the model.

The protocol was approved by the University of Southern Maine Institutional Review Board.

## 3. Results

During the study period (2000 to 2010), Maine PRAMS questionnaires were obtained from 12,600 women who gave birth to live infants. The response rate in Maine is consistently >70%. A total of 39 questionnaires were excluded from analysis due to unknown birth weights resulting in 12,561 questionnaires for analysis. For each logistic regression analysis we considered only pregnancy records for which all variables were available. In all cases this resulted in analysis of >10,400 pregnancies.

### 3.1. Average Demographics, Health Behaviors, and Outcomes

We have previously published detailed mean demographic values, health behavior indicators, and outcomes for this dataset [18]. Briefly, the mothers in this study had a mean age of 28.1 years and a mean BMI of 25.8; 63.6% were married, 45.2% had no education past high school, 31.8% lived in households with incomes <$20,000/year, 96.9% were white, 42.5% had gestational weight gain above the recommended range, 63.1% drank alcohol prior to pregnancy, and 31.6% smoked tobacco prior to pregnancy. For outcomes, 8.1% of infants in this study were born at a gestational age <37 weeks, 5.7% had a low birth weight (<2500 gms), 13.2% had an excessive birth weight (>4000 gms), and 9.3% were admitted to an intensive care unit.

### 3.2. Prepregnancy BMI and Gestational Weight Gain as Outcome Predictors

Predictors of the negative infant outcomes in this study including admission to an intensive care unit, longer hospital stay, low and excessive birth weight, and premature birth are shown in Tables 1 [Table tab2] [Table tab3] [Table tab4]–5. Predictors of a negative maternal outcome (hypertension during pregnancy) are shown in Table 6.

Excessive prepregnancy weight and gestational weight gain predicted a range of negative outcomes for both mother and infant. Unsurprisingly, both a higher prepregnancy BMI and a gestational weight gain greater than recommended predicted maternal hypertension during pregnancy, as does smoking before pregnancy (Table 6). A higher prepregnancy BMI also predicted an increased risk of the infant having a longer hospital stay (Table 2), being born with an excessive birth weight (>4000 gms) (Table 4), and being born prematurely (<37 weeks of gestational age) (Table 5). A gestational weight gain greater than recommended predicted a longer hospital stay for the infant (Table 2) and excessive birth weight (>4000 gms) (Table 4).

A low prepregnancy BMI or a gestational weight gain less than the recommended range also correlated with some risks. The risk of an infant being admitted to an ICU is greater if the mother has a low prepregnancy BMI or a level of gestational weight gain less than recommended (Table 1). Women with gestational weight gains below the recommended range are also more likely to give birth to low birth weight infants (<2500 gms) (Table 3) and premature infants (<37 wks of gestation) (Table 5). Interestingly, having a gestational weight gain greater than recommended reduced the risk of giving birth to an underweight (Table 3) or premature (Table 5) infant.

### 3.3. Measures of Socioeconomic Status as Outcome Predictors

This study included 2 measures of socioeconomic status, household income, and educational attainment, as independent variables; both were important predictors of negative outcomes. Mothers with no education past high school were more likely to give birth to infants who were admitted to an ICU (Table 1) or had low birth weight (Table 3). Women who lived in households with incomes <$20,000/year were more likely to give birth to infants who were admitted to an ICU (Table 1), had a longer hospital stay (Table 2), or were born prematurely (<37 wks of gestation) (Table 5). Maternal alcohol consumption reduces the risk of giving birth to an underweight (Table 3) or premature infant (Table 5) while smoking before and during pregnancy reduced the risk of giving birth to an infant with excessive weight (Table 4). However, given the well-documented risks of alcohol consumption and smoking during pregnancy, these findings are hardly an endorsement of these behaviors.

## 4. Discussion

### 4.1. Risks of High Prepregnancy BMI and Excessive GWG

The findings reported here showing that increasing prepregnancy BMI and excessive GWG are risks to both mother and newborn infant are in agreement with multiple previous studies [2–16, 22]. These issues are of substantial importance in Maine where prepregnancy BMI increased significantly for women who gave birth between 2000 and 2010 [20] and nationally where the Healthy People 2020 initiative has objectives of increasing the proportion of women delivering a live infant who had a healthy weight prior to pregnancy and increasing the proportion of mothers who achieve a recommended weight gain during their pregnancies [23]. How should these important health issues be addressed?

The US Department of Health and Human Services encourages primary care providers to talk to all of their patients about overweight/obesity [24]. Furthermore, current guidelines recommend that primary care providers screen all patients for overweight/obesity using the calculated BMI and institute comprehensive high-intensity interventions for obese patients [25]. These recommendations are particularly important for women who are planning to become pregnant, a group for whom weight reduction counseling is deemed a very high priority [26, 27]. Although lifestyle changes are always difficult to institute and maintain, women who are planning to become pregnant may be receptive to making positive lifestyle changes out of concern for the health of their infant [28]. Thus, pregnancy may present an optimal opportunity to counsel overweight women in minimizing gestational weight gain and promote lifestyle changes that will result in long-term weight management.

Once a woman becomes pregnant, providers of prenatal care should endeavor to meet the guidelines developed by the Institute of Medicine (IOM) and endorsed by the American College of Obstetricians and Gynecologists (ACOG) for nutrition and weight gain counseling [14]. Counseling about weight, nutrition, and physical activity should be ongoing throughout the pregnancy, interventions which have been found effective at helping women achieve a healthy level of gestational weight gain (GWG) [29]. Unfortunately, most healthcare providers give limited advice to overweight pregnant women about healthy gestational weight gain, proper diet, and physical activity [30]. This may be the result of lack of time during visits [31]; lack of proper knowledge and training about the topic [32]; disrupted or inadequate counseling [33]; and lack of patient comprehension of presented information [34]. A group prenatal care model known as “Centering Pregnancy” [35] has been proposed as a method to improve gestational weight gain and found to be effective in some [27] but not all [36] studies.

Prior to pregnancy, obese women who are not achieving optimal weight through lifestyle management may benefit from medical interventions including behavioral therapy, pharmacotherapy, and bariatric surgery. Weight loss medications can be useful adjuncts to lifestyle changes in patients with a BMI greater than 30 kg/m^2^ who have failed to achieve weight loss goals. However, the role of weight loss medications in obesity management remains controversial because of the side effects of these medications and concerns about long-term efficacy. There are five drugs approved for weight loss management in the United States; however none of these drugs are approved for use during pregnancy. Current guidelines recommend that weight loss drugs be discontinued if the patient exhibits any adverse effects or does not achieve adequate weight loss. The definition of adequate weight loss varies among medications but is generally defined as 5 percent or more over baseline within 3 to 6 months [37].

Pregnant women who have no contraindications should be advised to participate in physical activity, as recommended by the US Department of Health and Human Services guidelines, of at least 150 minutes of moderate-intensity physical activity, such as brisk walking, hiking, or bicycling, per week [38]. There is evidence that exercise during pregnancy can control weight gain and improve pregnancy outcomes. Several studies, including randomized controlled trials, have demonstrated that exercise inversely affects weight gain during pregnancy [39–41]. Physical activity during pregnancy may have additional benefits as demonstrated in a retrospective study that found pregnant women who were physically active during pregnancy spent less time in labor, an improvement that was more pronounced in multiparous women [42].

### 4.2. Benefits of High Prepregnancy BMI and Excessive GWG?

This study also found what appear to be benefits of having a higher prepregnancy BMI or GWG. Both a higher prepregnancy BMI and a GWG > recommended decrease the risk that the infant will be admitted to an ICU (Table 1), and a GWG > recommended also decreases the risk that an infant will be born premature by dates or weigh <2500 gms at birth (Tables 3 and 5). While these findings do not counterbalance the negative impacts of higher prepregnancy BMI or GWG found in this and many other studies (Tables 2, 4, 5, and 6) they do raise the issue of how a healthy BMI and GWG are determined.

The issue of how BMI impacts health is complex. A meta-analysis of international scope found that obesity is associated with increased all-cause mortality. It also found that people who are overweight but not obese exhibit decreased mortality compared to those of “normal” weight [43]. Similar results were obtained in a Canadian study, which found that overall mortality rates increased at BMI values <25 for women and <26 for men and that adiposity was a better predictor of mortality risk than was BMI [44]. Interestingly, the BMI associated with the lowest all-cause mortality seems to have increased since the mid-1970s, at least in Denmark [45].

Previous studies have also found that, along with its positive impacts, stringent control of GWG in obese women may increase risk of prematurity, low birth weight, and infant admission to an ICU [46], results in agreement with the dangers of inadequate GWG reported here (Tables 1, 2, 3, and 5). We also found that GWG > recommended decreases the risk that an infant will be born before 37 weeks of gestation or weighing <2500 gms. However, the positive correlation between maternal prepregnancy BMI and infant birth weight is well established [47] and may even have a generic basis in some individuals [48]. Thus our findings may reflect the fact that women who have a low BMI prior to conception have a higher risk of having small and premature infants [48] (Table 5) but would have to gain much more weight during pregnancy to have a gestational weight gain greater than the IOM recommended range [14].

### 4.3. Benefits of GWG Less Than Recommended?

Our results show that, overall, having a GWG < IOM recommended amounts increased the risk of poor outcomes. When compared to women with GWG within the recommended range, women who had a GWG < recommended were more likely to give birth to infants who were admitted to the ICU, weighed <2500 gms, and were born prematurely (Tables 1, 3, and 5). However, we also found that women who had a GWG < IOM recommended amounts were less likely to give birth to an infant with macrosomia (Table 4). The reason for this finding is not known. However, some previous studies have found that women with a prepregnancy BMI in the obese range who have very low GWG appeared to obtain some benefits.

A pilot study of obese women with type 2 diabetes mellitus conducted in Denmark compared women who had a GWG < 5 kg (<than the IOM recommended) to women who had GWG > 5 kg. Women with GWG < 5 kg in this study were less likely to give birth to large for gestational age infants, although not less likely to give birth to infants weighing >4000 gms [49]. A much larger study of obese pregnant women with and without diabetes found that women who gained <5 kg or even lost weight during pregnancy reduced their risk of developing gestational hypertension, requiring an emergency cesarean section, or giving birth to an infant with macrosomia, with no increase in the risk of giving birth to an infant with low birth weight, compared to women with higher GWG [50]. Further study of the impact of GWG for specific patient populations is important to allow for the appropriate evolution of IOM recommendations.

### 4.4. Impact of Socioeconomic Status (SES) on Pregnancy Outcomes

SES can be defined by occupation, education, or income, and marital status is a related variable [51]. Lower SES is a general health risk; both men and women in the US with lower incomes have lower life expectancies [52]. However, the mechanism of how SES impacts health is not easy to determine. For instance, greater educational attainment correlates with both improved health and higher income, but the known positive impact of higher income on health explains only part of the improvement in health as income increases. This suggests that greater educational attainment may alter decision-making patterns in healthy ways [53].

The correlation between increased obesity risk and lower socioeconomic status in developed countries is well documented [52]. Biro et al. [53] found that adults living in impoverished neighborhoods in Canada had an obesity risk > 1/3 higher than the risk to adults living in more affluent neighborhoods. Similarly, Drewnowski et al. [54] found that lower income and education as well as residence in an impoverished neighborhood increased obesity risk for adults in Seattle, USA, and Paris, France, while Larder et al. [55] found that prepregnancy obesity was higher among women in Michigan with no more than a high school education giving birth to a live infant compared to women who were college graduates. The health risks of lower SES extend beyond obesity, however. Women with lower SES are also at high risk to drink alcohol and have either primary or secondary exposure to tobacco smoke [56].

Lower SES, obesity, tobacco exposure, and alcohol consumption can all impact pregnancy outcomes and interact in complex ways. Previous studies have shown that women who have lower SES and/or live in poor neighborhoods are at increased risk for giving birth to infants who are premature or low birth weight [57–60]. But some of that effect may be the result of smoking. Furthermore, lower SES is associated with prepregnancy obesity [55] which in turn is associated with excessively high birth weight [61], a correlation which might obscure the risk that lower SES poses of low birth weight and prematurity. The results we report here confirm the link between lower SES (as measured by income or educational attainment) on the one hand and the risk of the infant being born premature or having a low birth weight and also show that lower SES is a risk for the infant being admitted to the ICU and having a longer hospital stay (Tables 1, 2, 3, and 5). The fact that the risks we found for lower SES occurred in a multivariable analysis in which alcohol and tobacco use were also considered suggests that SES has a negative impact on pregnancy outcomes independent of these other risks. By contrast, we also found that increasing prepregnancy BMI correlated with higher risk of giving birth to an infant weighing >4000 gms in an analysis where lower SES actually decreased risk (Table 4). This underscores the direct importance of prepregnancy BMI in macrosomia risk.

### 4.5. Limitations and Conclusions

This study has the limitations inherent in the PRAMS dataset. PRAMS questionnaires collect self-reports, which can be unreliable in matters such as weight, weight gain, and health behaviors. Furthermore PRAMS data include only women who have delivered a live-born infant and thus do not capture women whose pregnancies ended in a miscarriage, fetal death, or stillbirth.

This study also has limitations that arise from decisions made during data analysis. In our data analysis we could have excluded women with gestational diabetes mellitus (GDM) but chose not to do so. Women with GDM commonly have other important risk factors such as older age, higher prepregnancy BMI, and greater GWG [62], all variables we did include and found to be significant predictors of 1 or more adverse outcomes in our analysis. Thus excluding women with GDM would have also excluded women with these risk factors and weakened our ability to show their significance. Nonetheless, it is important to recognize that GDM, which may be an independent risk for adverse outcomes such as macrosomia [63], was not included in this analysis.

There are also multiple methods of categorizing birth weight; birth weight can be categorized into fixed weight categories or, taking gestational age into account, can be categorized as large for gestational age (LGA) or small for gestational age (SGA). We used the fixed birth weight categories of <2500 gms, 2500–3999 gms, and ≥4000 gms because this is the system used by the National Institutes of Health [20]. We also used a birth weight of <2500 gms for low birth weight rather than SGA because this is the definition of low birth weight used in the objective of Healthy People 2020 [64] and a birth weight of ≥4000 gms to identify infants with macrosomia rather than LGA because this is the most stringent definition used by the American College of Obstetricians and Gynecologists [65]. Furthermore, studies with large sample sizes such as the one we report here that use both systems to define infants who are too large or too small at birth commonly find that the two methods yield at least qualitatively similar results [50].

Nonetheless, our results report strong correlations between prepregnancy BMI, gestational weight gain, and SES (among other variables) and negative pregnancy outcomes infant admission to an ICU, longer hospital stays by infants, prematurity, and both low and excessive birth weight. These results highlight which women are at risk to have negative pregnancy outcomes. They underscore the importance of high-quality preconception care to ensure that a woman is in optimal health prior to becoming pregnant. Because not all of the risk variables identified in this study are directly modifiable by the healthcare system (e.g., lower SES) healthcare providers would do well to focus on patient education around modifiable risk factors such as eating habits, physical activity, and smoking cessation in all women of childbearing age who plan to become pregnant. Once a woman is pregnant education around GWG should also be addressed.

## Figures and Tables

**Table 1:** Maternal predictors of infant admission to ICU.

	Odds ratio (95% CI)	*P*
Smoked before pregnancy only/never smoked	0.992 (0.790–1.247)	0.9477
Smoked before and during pregnancy/never smoked	0.923 (0.743–1.146)	0.4656
Age	1.001 (0.986–1.016)	0.8741
First live birth/previous live birth	1.454 (1.249–1.692)	**<0.0001**
Not married/married	1.135 (0.939–1.373)	0.1908
Education ≤ 12 yrs/> 12 yrs	1.227 (1.034–1.457)	**0.0193**
Prepregnancy BMI	0.974 (0.965–0.983)	**<0.0001**
Annual HH income ≤ $20 k/> $20 k	1.313 (1.074–1.606)	**0.0079**
Urban or suburban/rural town or isolated rural	1.540 (1.333–1.780)	**<0.0001**
Nonwhite/white	1.327 (0.863–2.039)	0.1973
Drank alcohol prior to pregnancy/did not drink alcohol	1.149 (0.987–1.338)	0.0736
Drank alcohol in last 3 months of pregnancy/did not drink	0.952 (0.707–1.281)	0.7449
Gestational age when being sure she is pregnant	0.989 (0.970–1.008)	0.2634
Gestational age at first prenatal visit	1.010 (0.987–1.034)	0.3768
Pregnancy weight gain < recommended/recommended	1.261 (1.064–1.495)	**0.0074**
Pregnancy weight gain > recommended/recommended	0.764 (0.645–0.905)	**0.0018**

Logistic regression results with infant admission to an intensive care unit as the dependent variable: the infant is more likely to be admitted to an ICU if the mother was having her first birth, had no education past high school, lived in a household with an annual income < $20,000/year, lived in an urban or suburban area, or had a gestational weight gain < recommended range (compared to within recommended range). The infant was less likely to be admitted to an ICU if the mother had a higher prepregnancy BMI or a pregnancy weight gain > recommended range (compared to within recommended range).

**Table 2 tab2:** Maternal predictors of longer hospital stay by newborn.

Comparison	Odds ratio (95% CI)	*P*
Smoked before pregnancy only/never smoked	1.141 (0.991–1.313)	0.0668
Smoked before and during pregnancy/never smoked	1.137 (0.987–1.308)	0.0745
Age	1.033 (1.023–1.043)	**<0.0001**
First live birth/previous live birth	1.626 (1.477–1.789)	**<0.0001**
Not married/married	1.105 (0.979–1.248)	0.1069
Education ≤ 12 yrs/> 12 yrs	0.923 (0.827–1.031)	0.1560
Prepregnancy BMI	1.035 (1.028–1.042)	**<0.0001**
Annual HH income ≤ $20 k/> $20 k	1.307 (1.151–1.486)	**<0.0001**
Urban or suburban/rural town or isolated rural	0.962 (0.879–1.054)	0.4074
Nonwhite/white	0.787 (0.596–1.040)	0.0921
Drank alcohol prior to pregnancy/did not drink alcohol	0.909 (0.824–1.004)	0.0593
Drank alcohol in last 3 months of pregnancy/did not drink	0.961 (0.800–1.155)	0.6737
Gestational age when being sure she is pregnant	0.999 (0.984–1.014)	0.9104
Gestational age at first prenatal visit	0.995 (0.981–1.009)	0.4786
Pregnancy weight gain < recommended/recommended	1.066 (0.943–1.205)	0.3046
Pregnancy weight gain > recommended/recommended	1.124 (1.015–1.107)	**0.0248**

Logistic regression results with length of infant hospitalization as the dependent variable: infants were more likely to spend longer time in the hospital if mother was older, was having her first birth, had a higher prepregnancy BMI, lived in a household with an annual income < $20,000/year, or had a gestational weight gain > recommended range (compared to within recommended range).

**Table 3 tab3:** Maternal predictors of low (<2500 gms) versus normal birth weight.

Comparison	Odds ratio (95% CI)	*P*
Smoked before pregnancy only/never smoked	1.113 (0.961–1.289)	0.1529
Smoked before and during pregnancy/never smoked	1.624 (1.424–1.853)	**<0.0001**
Age	1.033 (1.023–1.044)	**<0.0001**
First live birth/previous live birth	1.832 (1.658–2.024)	**<0.0001**
Not married/married	1.198 (1.058–1.356)	**0.0043**
Education ≤ 12 yrs/> 12 yrs	1.176 (1.055–1.311)	**0.0034**
Prepregnancy BMI	1.004 (0.997–1.011)	0.2993
Annual HH income ≤ $20 k/> $20 k	0.895 (0.787–1.017)	0.0882
Urban or suburban/rural town or isolated rural	0.970 (0.885–1.063)	0.5119
Nonwhite/white	1.089 (0.824–1.439)	0.5490
Drank alcohol prior to pregnancy/did not drink alcohol	0.838 (0.758–0.926)	**0.0005**
Drank alcohol in last 3 months of pregnancy/did not drink	0.799 (0.657–0.972)	**0.0248**
Gestational age when being sure she is pregnant	1.020 (1.004–1.036)	**0.0118**
Gestational age at first prenatal visit	0.972 (0.957–0.987)	**0.0003**
Pregnancy weight gain < recommended/recommended	2.161 (1.935–2.413)	**<0.0001**
Pregnancy weight gain > recommended/recommended	0.721 (0.646–0.804)	**<0.0001**

Logistic regression results with infant birth weight < 2500 gms as the dependent variable: compared to normal weight infants, infants are more likely to be born weighing < 2500 gms if their mother was older, was having her first child, was not married, had no education past high school, was not sure she was pregnant until later in gestation, had a weight gain < recommended range (compared to within recommended range), or smoked before and during pregnancy. Infants were less likely to be underweight if mother had a gestational weight gain > the recommended amount of weight (compared to within recommended range), had her first prenatal visit later in gestation, or drank alcohol before or during pregnancy.

**Table 4 tab4:** Maternal predictors of excessive (≥4000 gms) versus normal birth weight.

Comparison	Odds ratio (95% CI)	*P*
Smoked before pregnancy only/never smoked	0.933 (0.752–1.159)	0.5335
Smoked before and during pregnancy/never smoked	0.445 (0.338–0.586)	**<0.0001**
Age	0.999 (0.984–1.015)	0.9480
First live birth/previous live birth	0.677 (0.583–0.786)	**<0.0001**
Not married/married	1.089 (0.892–1.329)	0.4008
Education ≤ 12 yrs/> 12 yrs	0.815 (0.684–0.971)	**0.0221**
Prepregnancy BMI	1.030 (1.020–1.041)	**<0.0001**
Annual HH income ≤ $20 k/> $20 k	1.161 (0.934–1.444)	0.1783
Urban or suburban/rural town or isolated rural	0.974 (0.846–1.121)	0.7097
Nonwhite/white	1.026 (0.626–1.681)	0.9204
Drank alcohol prior to pregnancy/did not drink alcohol	1.021 (0.876–1.189)	0.7902
Drank alcohol in last 3 months of pregnancy/did not drink	1.150 (0.883–1.496)	0.2995
Gestational age when being sure she is pregnant	1.005 (0.981–1.030)	0.6675
Gestational age at first prenatal visit	1.011 (0.990–1.032)	0.3152
Pregnancy weight gain < recommended/recommended	0.723 (0.568–0.921)	**0.0087**
Pregnancy weight gain > recommended/recommended	2.210 (1.886–2.589)	**<0.0001**

Logistic regression results with infant birth weight > 4000 gms as the dependent variable: compared to normal weight infants, infants are more likely to be born weighing > 4000 gms if their mother had a higher BMI or a gestational weight gain > recommended range. Babies were less likely to have a birth weight > 4000 gms if their mother smoked before and during pregnancy, was having her first live birth, had no education past high school, or had a gestational weight gain < recommended range.

**Table 5:** Maternal predictors of infant born prematurely (<37 wks of gestation).

Comparison	Odds ratio (95% CI)	*P*
Smoked before pregnancy only/never smoked	0.925 (0.736–1.161)	0.5008
Smoked before and during pregnancy/never smoked	1.010 (0.823–1.239)	0.9261
Age	1.017 (1.002–1.032)	**0.0227**
First live birth/previous live birth	1.425 (1.230–1.650)	**<0.0001**
Not married/married	1.046 (0.873–1.254)	0.6238
Education ≤ 12 yrs/> 12 yrs	0.911 (0.769–1.079)	0.2802
Prepregnancy BMI	1.019 (1.009–1.028)	**0.0001**
Annual HH income ≤ $20 k/> $20 k	1.255 (1.032–1.524)	**0.0227**
Urban or suburban/rural town or isolated rural	0.884 (0.772–1.012)	0.0740
Nonwhite/white	1.341 (0.825–2.178)	0.2360
Drank alcohol prior to pregnancy/did not drink alcohol	0.787 (0.680–0.911)	**0.0013**
Did not drink alcohol in last 3 months of pregnancy/drank	0.832 (0.621–1.116)	0.2192
Gestational age when being sure she is pregnant	1.025 (1.006–1.045)	**0.0101**
Gestational age at first prenatal visit	0.972 (0.949–0.996)	**0.0217**
Pregnancy weight gain < recommended/recommended	1.645 (1.401–1.931)	**<0.0001**
Pregnancy weight gain > recommended/recommended	0.721 (0.610–0.853)	**0.0001**

Logistic regression results with infant being born at < 37 weeks of gestation as the dependent variable. Infants were more likely to be born at < 37 weeks of gestational age which is greater for mothers who were older, were having their first birth, had a higher prepregnancy BMI, lived in a household with an annual income < $20,000/year, were sure they were pregnant at a later gestational age, or had a gestational weight gain < recommended range. Infants were less likely to be born at < 37 weeks of gestational age if their mother drank alcohol before pregnancy, has her first prenatal visit at an earlier gestational age, or had a gestational weight gain <> recommended range.

**Table 6:** Maternal predictors of hypertension during pregnancy.

Comparison	Odds ratio (95% CI)	*P*
Smoked before pregnancy only/never smoked	1.205 (1.022–1.420)	**0.0260**
Smoked before and during pregnancy/never smoked	1.028 (0.866–1.220)	0.7558
Age	0.999 (0.987–1.011)	0.8272
First live birth/previous live birth	0.657 (0.586–0.736)	**<0.0001**
Not married/married	0.976 (0.840–1.133)	0.7458
Education ≤ 12 yrs/> 12 yrs	0.976 (0.855–1.113)	0.7140
Prepregnancy BMI	1.046 (1.037–1.055)	**<0.0001**
Annual HH income ≤ $20 k/> $20 k	1.018 (0.872–1.189)	0.8223
Urban or suburban/rural town or isolated rural	0.989 (0.888–1.102)	0.8456
Nonwhite/white	0.943 (0.673–1.320)	0.7307
Drank alcohol prior to pregnancy/did not drink alcohol	1.010 (0.898–1.135)	0.8742
Drank alcohol in last 3 months of pregnancy/did not drink	1.060 (0.849–1.323)	0.6070
Gestational age when being sure she is pregnant	0.993 (0.974–1.012)	0.4463
Gestational age at first prenatal visit	1.012 (0.995–1.029)	0.1582
Pregnancy weight gain < recommended/recommended	0.925 (0.792–1.081)	0.3293
Pregnancy weight gain > recommended/recommended	1.359 (1.205–1.534)	**<0.0001**

Logistic regression results with maternal hypertension as the dependent variable: mothers were more likely to be hypertensive during pregnancy if they smoke before pregnancy, had a higher prepregnancy BMI, or had a gestational weight gain > recommended as compared to within the recommended range. Mothers were less likely to be hypertensive if they were having their first live birth.
